# Using Wearable Sensor Technology to Measure Motion Complexity in Infants at High Familial Risk for Autism Spectrum Disorder

**DOI:** 10.3390/s21020616

**Published:** 2021-01-17

**Authors:** Rujuta B. Wilson, Sitaram Vangala, David Elashoff, Tabitha Safari, Beth A. Smith

**Affiliations:** 1 Semel Institute for Neuroscience and Human Behavior, David Geffen School of Medicine, University of California Los Angeles, 760 Westwood Plaza, Los Angeles, CA 90095, USA; BSafari@mednet.ucla.edu; 2 Department of Medicine Statistics Core, David Geffen School of Medicine, 1100 Glendon Avenue, Suite 1820, Los Angeles, CA 90024, USA; SVangala@mednet.ucla.edu (S.V.); DElashoff@mednet.ucla.edu (D.E.); 3 Developmental Neuroscience and Neurogenetics Program, The Saban Research Institute，Division of Research on Children, Youth, and Families, Children’s Hospital Los Angeles, Department of Pediatrics, Keck School of Medicine, University of Southern California, Los Angeles, CA 90089, USA; bsmith@chla.usc.edu

**Keywords:** motor development, autism spectrum disorder, wearable sensors, quantitative measures

## Abstract

Background: Motor dysfunction has been reported as one of the first signs of atypical development in infants at high familial risk for autism spectrum disorder (ASD) (HR infants). However, studies have shown inconsistent results regarding the nature of motor dysfunction and whether it can be predictive of later ASD diagnosis. This is likely because current standardized motor assessments may not identify subtle and specific motor impairments that precede clinically observable motor dysfunction. Quantitative measures of motor development may address these limitations by providing objective evaluation of subtle motor differences in infancy. Methods: We used Opal wearable sensors to longitudinally evaluate full day motor activity in HR infants, and develop a measure of motion complexity. We focus on complexity of motion because optimal motion complexity is crucial to normal motor development and less complex behaviors might represent repetitive motor behaviors, a core diagnostic symptom of ASD. As proof of concept, the relationship of the motion complexity measure to developmental outcomes was examined in a small set of HR infants. Results: HR infants with a later diagnosis of ASD show lower motion complexity compared to those that do not. There is a stronger correlation between motion complexity and ASD outcome compared to outcomes of cognitive ability and adaptive skills. Conclusions: Objective measures of motor development are needed to identify characteristics of atypical infant motor function that are sensitive and specific markers of later ASD risk. Motion complexity could be used to track early infant motor development and to discriminate HR infants that go on to develop ASD.

## 1. Introduction

In the first year of life, infants dynamically develop motor skills and achieve major motor milestones such as rolling, crawling, and walking. The earliest motor skills, such as spontaneous leg movements, emerge and evolve in a way that lays the framework for locomotion [1,2,3]. These motor milestones are fundamental areas that caregivers and practitioners can visibly evaluate to determine typical progression of development. Motor development allows an infant to (1) build social and environmental experiences that support cognitive and perceptual skills, (2) receive sensory input from exploration of different surfaces and anti-gravity positions, and (3) develop a repertoire of complex motor behaviors through movement mistakes and achievements [4,5,6,7,8]. Conversely, dysfunctional motor development can have a negative cascading effect on cognition, spatial and physical perception, and appropriate development of higher-level motor abilities such as coordination and balance [6,7,9,10,11]. There are several neurodevelopmental disorders that have associated findings of gross motor dysfunction, including autism spectrum disorder (ASD), attention deficit/hyperactivity disorder, and intellectual disability [12,13,14,15,16,17]. In ASD, motor dysfunction has been posited to be one of the earliest signs of risk for later diagnosis [18,19]. Studies of infants at high familial risk for ASD (defined as infants with a sibling with ASD and often termed “HR infants”), have described findings of head lag and delays in sitting and standing in the first year of life. Repetitive motor movements, a core diagnostic criteria for ASD, have been described in some studies to be apparent at 12 months of age [12,20,21]. For these reasons, there is interest in identifying when motor differences first emerge, whether these differences can be predictive of later a neurodevelopmental disorder (NDD) diagnosis, and whether they are amenable to intervention.

Currently, measurement of infant motor development relies on brief observations using standardized motor assessments. These motor assessments have many potential limitations including: (1) evaluations are based on subjective visual observation, (2) scoring is often binary in nature, (3) infants may not perform their full motor repertoire when examined in different settings (e.g., clinic vs. the home), and (4) there is a wide range of motor progression in typical development (e.g., the onset of independent sitting can range from 4–9 months of age). All of these limitations can make it difficult to distinguish motor differences [22,23]. These limitations have also hampered motor studies of HR infants in which there has been an array of results that describe the nature of motor differences and whether they are predictive of a later ASD diagnosis [24]. Although these studies have provided important knowledge to the field, it is likely that current standardized motor assessments are not able to identify subtle motor impairments that may precede and underlie the heterogeneous observable motor dysfunction that has been reported. There has been little use of objective measures of infant movement, such as wearable sensor technology, to study motor development in infants at high familial risk for ASD. Wearable sensors are quantitative, objective, and can overcome some of the limitations of current standardized motor assessments. Importantly, wearable sensors can be used in the home environment and identify more detailed characteristics of early infant motor development such as movement type, frequency, and duration [25].

In the present study we utilized wearable sensors to measure movement in infants at high familial risk for ASD (hereafter, “HR infant”) in the first year of life. From the quantitative sensor data, we specifically chose to develop a measure of motion complexity. We focus on complexity of motion because we follow the theoretical perspective that healthy neuromotor function is associated with a state of maximum complexity [26]. It has been described that lack of complex and variable movement leads to abnormal mapping of the sensory cortex, which can in turn disturb motor function [26,27]. Lower complexity of motion has also been described in infants at risk for cerebral palsy and infants broadly at risk for developmental delays [23,26,27,28,29]. Thus, low complexity of infant motion could indicate atypical development and underlie later observable motor delays. Another aspect of complexity of movement is repeatability of a movement signal; lower complexity may indicate more repeatable and less variable movements.

The creation of a motion complexity measure is both novel and theoretically relevant in the study of motor development in infants at high familial risk for ASD. First, because lower complexity of motion could underlie the later observable heterogenous motor delays and second, because it could represent the presence of atypical repetitive motor behaviors in early infancy. In turn, this measure could be utilized to predict which infants and toddlers might go on to have a greater burden of stereotyped motor behaviors, a core diagnostic feature of ASD. In this index development study, we first describe in detail the creation of this novel measure of infant motion complexity. As proof of concept, we then test the hypothesis that lower motion complexity relates to atypical developmental outcomes by examining the relationship of the complexity measure to behavior and developmental measures in a small set of HR infants. We hypothesize that HR infants that go on to develop ASD will show lower movement complexity compared to those who do not. The necessary next steps will be to translate the use of motor complexity to a larger sample size of infants at high familial risk for ASD and other developmental delays that are known to have associated motor abnormalities. The ultimate goal of this work is to develop quantitative motor parameters that can provide early identification of ASD and mechanistic insight on why motor delays occur and how they affect downstream development.

## 2. Materials and Methods

Developmental data were collected as a part of a prospective longitudinal study of early brain and behavioral markers of ASD at the University of California, Los Angeles (UCLA) (ACE P50HD055784-11). Motor data were collected as a part of a prospective longitudinal study of infant leg movement patterns at the University of Southern California (USC).

### 2.1. Procedures

Ethical approval was obtained by the Institutional Review Boards of UCLA and USC, and one parent or legal guardian signed an informed consent form for each study prior to participation. Infants were enrolled in the HR group if they had at least one older sibling with a confirmed clinical diagnosis of ASD. ASD diagnoses were confirmed by the principal investigator by review community or clinical reports of ASD evaluation and diagnosis. HR infants were excluded if they had a genetic syndrome (e.g., Tuberous Sclerosis Complex, Down Syndrome, Fragile X Syndrome). The infants included in this study completed both wearable sensor data collection and developmental outcome assessments at 36 months of age. Developmental assessments took place at the UCLA Center for Autism Research and Treatment. All developmental assessments were conducted by trained research reliable clinical psychologists or psychometrists. The motor assessments were conducted in the participants’ homes.

#### 2.1.1. Quantitative and Standardized Motor Assessment

**Figure 1:**
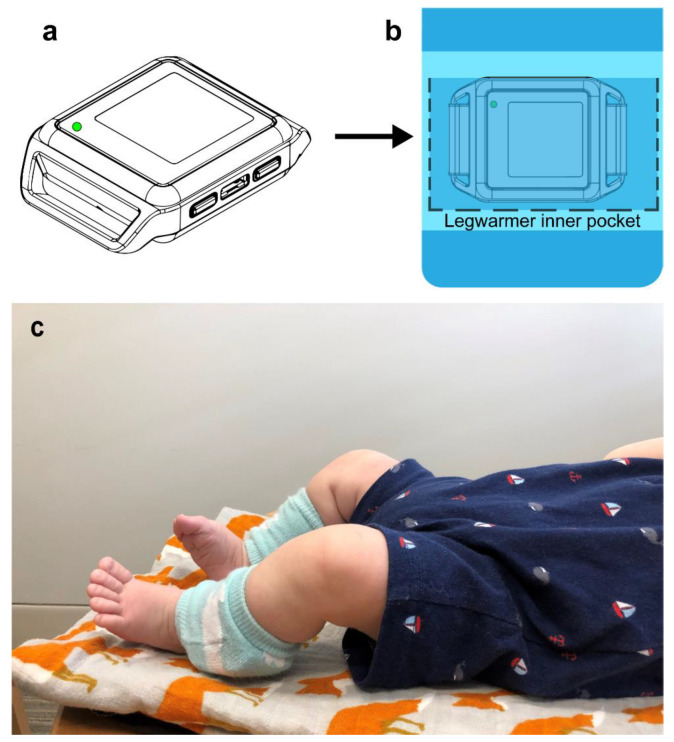
(**a**) Illustration of a wearable sensor; (**b**) Illustration of how the wearable sensor is positioned within a legwarmer; (**c**) Infant wearing sensors and legwarmers on both ankles.

Utilizing the wearable sensors, leg movement data from were collected from five HR infants at 3, 6, 9, and 12 months of age. The protocol included visitation of infants each morning in their home environment with the goal of collecting full day movement data. The wearable sensors were placed on the infants’ ankles and were attached using legwarmers. Each ankle legwarmer contains a pocket to hold the wearable sensor in place as shown in Figure 1a–c. Families were instructed to go about their typical daily activities. The infants wore the sensors until bedtime, resulting in a total of 8–12 h of data. During each visit, infants’ measurements (weight, length, and head circumference) were measured. Motor development status was quantified using the Alberta Infant Motor Scale (AIMS). The AIMS assesses gross motor skills from 0–18 months of age and includes four positional subscales (prone, supine, sit, and stand) each yielding a subscale score. The sum of the positional subscales yields the infant’s total score. A percentile score is then derived based on the infant’s age and total score [30]. The characteristics of the HR infants are summarized in Table 1.

#### 2.1.2. Developmental Evaluation

The Autism Diagnostic Observation Schedule-Second version [ADOS] [31] is an observational measure of social-communication and repetitive behaviors and this measure was used to evaluate ASD symptoms at 18 and 36 months of age. At 18 months of age the ADOS toddler module was used, and the outcomes are measured as level of concern for ASD (little to no concern, mild to moderate concern, and moderate to severe concern). At 36 months of age the ADOS module 1 was used for ASD evaluation, and this time point was used as the final diagnostic outcome.

The Mullen Scales of Early Learning [MSEL] [32] was used to assess cognitive level at 36 months of age. The MSEL examines fine motor, visual reception, receptive language, and expressive language and each domain yields T-scores (mean (SD), 50 (10)). From the subscales an early learning composite (ELC) is also calculated, yielding a standard score (mean (SD), 100 (15)). The ELC represents a child’s overall cognitive ability relative to peers. The ELC composite is displayed in Table 2.

The Vineland Adaptive Behavior Scale—Second Edition [VABS-II] [33] was used to assess adaptive skills at 36 months. The VABS-II is a semi-structured interview conducted with the parent and assesses four domains of adaptive behavior: (a) socialization, (b) daily living skills, (c) communication, and (d) motor skills. The adaptive behavior composite is computed from the first three domains, yielding a standard score representing an individual’s overall adaptive ability relative to peers. The Adaptive Behavior Composite score is displayed in Table 2.

#### 2.1.3. Sensor Data

APDM (Ambulatory Parkinson’s Disease Monitoring) Opal wearable sensors were used in this study and are comprised of 3D-accelerometer, 3D-gyroscope, and 3D-magnetometer. Opal sensors are wireless and lightweight and have been used to study infant movement [25,34,35]. The sensor acceleration range is 6 g, and measurements are reported with 14-bits resolution. Recordings were made at 20 hz. The data from both left and right sensors were actively synchronized throughout the recording, stored on the internal memory of each individual sensor, and downloaded at the end of each visit. The data were recorded continuously, and thus included sleep and awake states.

#### 2.1.4. Sensor Data Pre-Processing and Development of the Motion Complexity Index

Single infant leg movements were extracted using a validated algorithm [36]. The algorithm is threshold based and is able to distinguish separate leg movements when a leg pauses or changes direction. From the extracted leg movements, the following movement data is computed: duration of movement, peak acceleration, and average acceleration during a movement.

Using the full-day movement data derived from the sensor, we sought to construct a novel measure of motion complexity. A minimally complex movement pattern is one fully described by a singular periodic function (e.g., a sine wave with a specified frequency). High complexity, in contrast, indicates that motion is best described by superposing of waves of varying frequencies. We therefore defined our complexity measure in terms of the variability of the frequency components underlying the observed movements.

Fourier transformation was used to recover the frequency components, translating the movement data from the time domain into the frequency domain. The frequency domain represents a time series as a collection of amplitude-frequency pairs, representing each of the underlying frequency components. The superposition of waves represented by each component reconstructs the original time series. This translation was performed using the Fast Fourier Transform (FFT), a standard implementation widely used in time series analysis and signal processing [37].

To remove the influence of average magnitude of motion, as well as variability in the magnitude of motion over the course of the day, we standardized the full-day motion data using z-scoring prior to applying the FFT. While magnitude and time-variability may provide useful information about future ASD diagnosis probabilities, these are distinct aspects of motion patterns that might otherwise confound our complexity measure.

Because throughout the day the child transitions between sleep, waking, and active states, we divided the full day’s data into brief time segments, and only included segments with a minimum number of motions in the calculation of the measure, to ensure that we are focusing on active states when the data would be expected to be most informative. We then averaged the measure over the multiple segments per day to come up with a day-level measurement.

**Figure 2:**
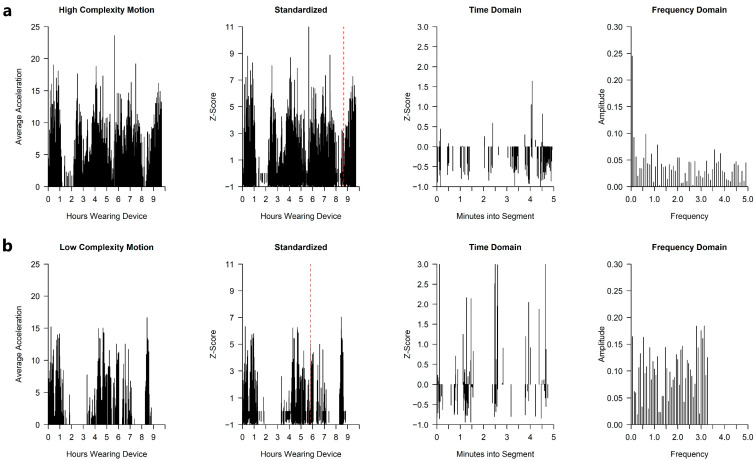
These figures display the steps taken to construct the motion complexity measure. (**a**) depicts an HR infant with high motion complexity, and (**b**) depicts an HR infant with low motion complexity. Column one is the raw, full-day average acceleration data for the left leg. Column two represents the data in column one after the data has been z-scored (overall magnitude and variability of motion are now comparable between both cases). Column three represents a high and low complexity 5-min time segment (indicated by red dashed line in column two). Column four represents the data from column three after it has been translated to the frequency domain. In column four, the components of the high-complexity motion vary more widely in frequency than the components of the low-complexity motion.

In summary, the following steps were used to construct a day-level complexity measure: (1) z-score the full-day motion data; (2) divide the day into brief, equal-length time segments; (3) discard segments with fewer than a minimum number of movements; (4) perform FFT on each segment; (5) compute the amplitude-weighted standard deviation of frequency components in each segment; (6) average these standard deviations across the included segments to produce the measure. Our specific implementation of the measure used the average acceleration data from the left leg, 5-min time segments, and a minimum of 50 movements per included segment. As a sensitivity analysis, we constructed alternative versions of the measure, using the right leg, using peak rather than average acceleration, using 2.5 or 10-min time segments, and using 25 or 100 as the minimum number of movements per segment, finding that these alternatives were very highly correlated with our preferred implementation, suggesting measure robustness. Figure 2 illustrates the steps of measure construction.

If each active segment of the motion signal is fully described by a singular wave pattern, then our measure will return a complexity score of 0. The more wave patterns combine, the more the frequencies of the constituent waves vary, and the more uniform the corresponding amplitudes, the higher the score will be. In this sense, the proposed algorithm closely aligns with the intended conception of movement complexity.

#### 2.1.5. Evaluation of Motion Complexity Score and Developmental Outcomes

We evaluated the relationship of the motion complexity score at each motor study time point (3, 6, 9, 12 months of age) to ASD concern at 18 months and ASD diagnosis at 36 months and cognitive ability and adaptive skills at 36 months. We also examined the relationship of change in the motion complexity score from baseline to 3, 6, and 9 months to developmental concerns and outcomes. We display the motion complexity data in relation to developmental concerns and outcomes using scatterplots, and we report Pearson correlation coefficients to summarize the observed patterns in these five HR infants. Due to the small sample size, we refrain from making any statistical inferences, and instead interpret these correlations descriptively.

## 3. Results

### 3.1. Motion Complexity Score

The motion complexity score at each visit time point showed a range of results in the five HR infants. The range of motion complexity score results by age is as follows: 3 months of age (0.373–0.676), 6 months of age (0.393–1.041), 9 months of age (0.382–1.027), and at 12 months of age (0.356–0.840). We did not find any specific pattern in change in complexity from 3 months to 6 months, 3 months to 9 months, and 3 months to 12 months of age (e.g., complexity increases or decreases with age).

### 3.2. Developmental Outcomes

At 18 months of age on the ADOS toddler module, two of the high-risk infants met criteria for moderate to severe concern for ASD, one infant met for mild to moderate concern for ASD, and two infants met for little to no concern for ASD. At 36 months of age on the ADOS module 1, two infants met criteria for ASD, and three infants did not. The infant that had mild to moderate concern for ASD at 18 months did not meet ASD diagnostic criteria at 36 months of age. However, for this infant, parents reported concerns for general development during the 36-month research evaluation. There was a range of cognitive abilities (MSEL ELC) and adaptive skills (Vineland ABC) in the HR infants regardless of ASD outcome (Table 2).

### 3.3. Relationship of the Motion Complexity Score and Developmental Outcomes

**Figure 3:**
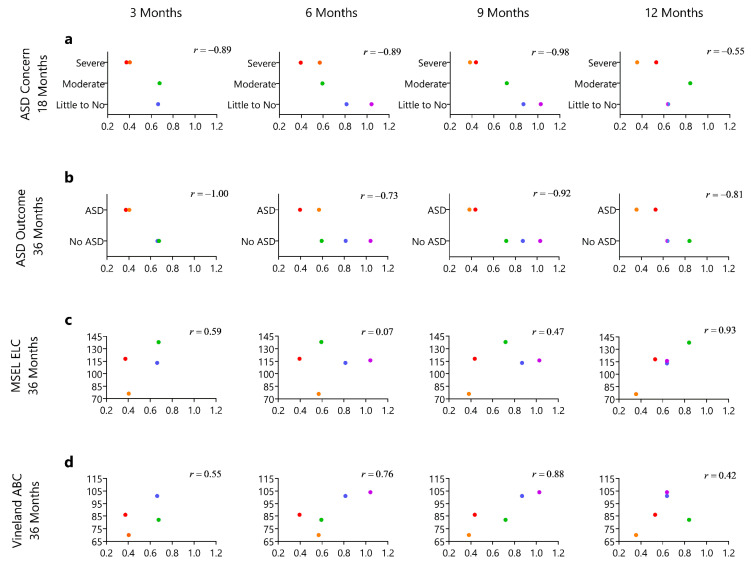
The following series of graphs represents the developmental outcome on the *y*-axis by visit level motion complexity score on the *x*-axis at 3, 6, 9, and 12 months of age. (**a**) The *y*-axis represents Autism Spectrum Disorder (ASD) concern at 18 months of age indicated by severe concern, moderate concern, and little to no concern. (**b**)The *y*-axis represents Autism Spectrum Diagnosis at 36 months of age (diagnostic outcome assessment) as indicated by diagnosis of ASD and No ASD. (**c**) The *y*-axis represents the cognitive ability as measured by the Mullen Scales of Early Learning (MSEL), Early Learning Composite. (**d**) The *y*-axis represents adaptive skills as measures by the Vineland Adaptive Behavioral Scale-II, Adaptive Behavior Composite.

**Figure 4:**
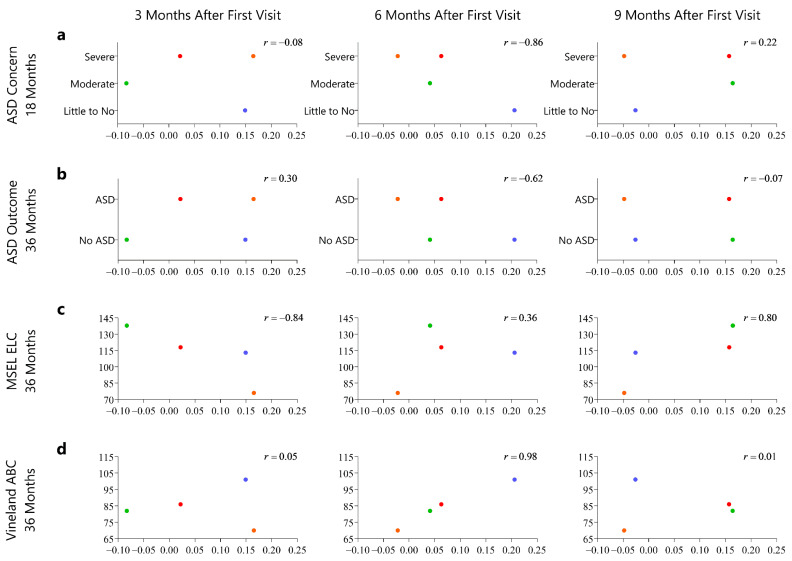
The following series of graphs represents developmental outcomes on the *y*-axis change in motion complexity score from 3–6, 3–9, and 3–12 months of age. (**a**) The *y*-axis represents Autism Spectrum Disorder (ASD) concern at 18 months of age indicated by severe concern, moderate concern, and little to no concern. (**b**) The *y*-axis represents Autism Spectrum Diagnosis at 36 months of age (diagnostic outcome assessment) as indicated by diagnosis of ASD and No ASD. (**c**) The *y*-axis represents the cognitive ability as measured by the Mullen Scales of Early Learning (MSEL), Early Learning Composite. (**d**) The *y*-axis represents adaptive skills as measures by the Vineland Adaptive Behavioral Scale-II, Adaptive Behavior Composite.

We found that the two infants that went on to develop ASD showed lower motion complexity scores compared to the three infants that did not. This difference in motion complexity scores between the infants was noted as early as three months of age and were observed for all the motor time points (Figure 3a,b). We observed some positive correlations between higher motion complexity score and higher cognitive ability and adaptive skills at the 36 month outcome assessments, but this was not consistent across all time points and correlations were generally not as strong (Figure 3c,d). The observed correlations with the change of motion complexity score and developmental concerns and outcomes were weaker and generally less consistent from time point to time point (Figure 4a–d).

## 4. Discussion

Here we present an index development study by developing a novel measure of motion complexity. We also lay the groundwork of utilizing wearable sensors to measure full day motor activity in HR infants. We take the first steps in establishing a theoretical model of the relationship of lower motion complexity to motor dysfunction and ASD. As proof of concept, we also provide very preliminary data that supports motion complexity may be useful to track early infant motor development and potentially discriminate HR infants that go on to develop ASD. In our small HR infant sample, there was no consistent relationship between motion complexity score and cognitive abilities and adaptive skills, or with change in complexity score and developmental outcomes. Given the prevalence of motor impairments in HR infants, the importance for early diagnosis, and the challenges associated with standardized motor assessments, a reliable and objective method of measuring clinically applicable infant motor development is needed.

Wearable sensors provide an unprecedented opportunity to capture an infant’s full movement repertoire in a more naturalistic environmental setting. Unlike brief motor assessments that occur in the clinic or research setting, we were able to collect full day movement data in the home environment. The data are quantitative and objective and provide detailed characteristics of infant motor development. We leveraged the use of the wearable sensor to create a hypothesis driven novel measure of motion complexity. We took specific steps in creation of the measure to ensure reliable representation of infant movement. In support of proof of concept, the two HR infants who developed ASD showed lower motion complexity at early ages. These data suggest that quantitative measures can aid in our ability to evaluate subtle motor differences in early infancy. Furthermore, it allows for the measurement of motor activity that plays an intrinsic role in the development of motor skills.

We chose specifically to measure motion complexity because we hypothesize that there is a mechanistic relationship between motion complexity and the atypical motor and behavioral manifestations of ASD. Our theoretical model rests on three areas: (1) the emerging evidence that complexity plays a key role in healthy neuromotor development, (2) the relationship of motion complexity and variability to repetitive motor behaviors, and (3) and the potential dual role of early atypical synaptogenesis in the neurobiology of motion complexity and ASD.

As early as in utero, the fetus displays general movements of all parts of the body. These spontaneous and self-generated movements continue into young infancy and two key features of healthy general movements are complexity and variation [38]. The theory that maximum complexity underlies healthy neuromotor maturation extends from infancy through adulthood. Complexity of motion is key for normal development of early infant motor skills such as spontaneous kicking, postural control, and gait. Lack of movement complexity in infants has been described as an indicator of cerebral palsy and has also been found in preterm infants and infants broadly at risk for developmental delays [23,28,39]. Thus, lack of motion complexity could be a precursor or an underlying cause of the later heterogeneous motor delays that have been described in HR infants.

Our complexity feature was created to capture variability of the underlying motion frequencies. Less complex motion indicates less variable or more repetitive motion, while more complex motion indicates greater variability and less repeatability of a motion. For that reason, we also hypothesize that measurement of motion complexity could detect repetitive motor behaviors in HR infants prior to 12 months of age. The identification of visible repetitive motor stereotypies at 12 months of age have shown promise in predicting later ASD diagnosis [21]. One example of this is a study that utilized the Repetitive Behavior Scales—Revised to longitudinally study HR infants from 12–24 months of age. They found that at 12 months of age, HR infants with a later diagnosis of ASD showed significantly higher rates of repetitive behaviors compared to LR infants [40]. Studies of early brain development have found that structural properties of callosal and cerebellar white matter pathways at six months of age were associated with later repetitive behaviors in autism, indicating that that aberrant neural circuitry contributing to these motor manifestations is present before the clinically observable trait [41]. Thus, it is possible that evaluation of infant movements prior to 12 months of age would allow for identification of atypical levels of repetitive movement signals. In our small sample of five HR infants, those that went on to develop ASD showed lower motor complexity as early as three months of age. We did not find the same relationship with motion complexity and cognitive abilities or adaptive skills. Repetitive motor behaviors are a core diagnostic criterion of ASD, and measurement of motion complexity could provide a more sensitive marker of those infants that will go on to develop ASD.

Lastly, there could be a dual neurobiological role in the development of atypical motion complexity and ASD. It has been described that the emergence of synaptic activity in the embryonic cortex coincides with the emergence of complex and variable movements, and in turn, the expression of motion complexity and variability depends on the integrity of cortical connectivity [27]. It has also been proposed that ASD is a disorder of synaptic plasticity that results in imbalances of excitation and inhibition in the developing brain [42,43,44]. Studies have shown aberrant cortical connectivity with an excess of short distanced cortical connectivity and a reduction of long distanced cortical connectivity [45,46]. If this is so, then it is possible that the evaluation of motion complexity can provide insight on which HR infants are more likely to develop ASD versus other atypical developmental outcomes (ADHD, ID, isolated social communication impairments). To test this hypothesis, it would be pertinent to examine the relationship of neuro-imaging and electrophysiological studies and motion complexity in early infancy. Quantitative measurement of motion complexity in HR infants can provide critical information for such studies.

### 4.1. Limitations

The goal of the present study was to develop an objective measure of infant motor development that could have clinical utility for early identification of ASD. While we established the first steps needed to develop this measure, there are limitations to this study that must be acknowledged. First, to draw any conclusions about the utility of this measure to diagnosis or predict ASD, it must be evaluated in a larger, longitudinal representative sample with ASD outcomes. Second, the objective measure needs to be validated against a gold standard measure of motion complexity at the time of data collection in a larger sample. Third, correlation of this measure to an appropriate measure of stereotyped movements would best test the hypothesis regarding the relationship of low motion complexity and higher repetitive motor behaviors. In addition, although we attempted to standardize the movement data, it is also possible that other motion characteristics such as number of movements could impact the motion complexity result. Thus, the results should be interpreted with caution until further steps can be taken to validate the motion complexity measure.

### 4.2. Next Steps

To improve interpretation of the use of this measure in the ASD population, we plan to evaluate the motion complexity measure in a larger longitudinal sample of HR infants and include infants at low familial risk for ASD as a comparison group. Future steps will also include comparison of HR infants with infants more broadly at risk for motor delays (e.g., prematurity, prenatal intracranial injury) to determine whether differences in motion complexity are specific to ASD. We also plan to evaluate the use of this measure compared to video-based expert observation and rating of complex infant movements. Lastly, moving forward, we plan to include a measure of repetitive motor behaviors at 12 and 24 months and compare these results to the motion complexity measure. These steps are necessary to both validate this measure and to draw any conclusions regarding the possible specificity of motion complexity in ASD.

## 5. Conclusions

The study of HR infants can yield crucial information on when motor impairments emerge in ASD, why they occur, and whether they are related to later development of ASD. To answer these questions, objective measures of motor development are needed to identify detailed characteristics of atypical infant motor performance that could be sensitive and specific markers of later ASD risk. In this study, we take the first steps in establishing a quantitative motor parameter that may be indicative of risk for ASD. This study provides preliminary evidence that motion complexity has potential to track early infant motor development and to discriminate HR infants that go on to develop ASD.

## Figures and Tables

**Table 1:** Infant Characteristics.

Visit in Months	N	Age (days)	Weight (kg)	Body Length (cm)	Head (cm)	AIMS Percentile
3	4	111 (32), (90–158)	7.375 (1.4), (5.7–8.9)	63.9 (5.0), (60.1–71.0)	41.8 (1.5), (39.8–43.2)	38 (20), (14–67)
6	5	198 (29), (180–250)	8.5 (1.9), (5.9–10.7)	67.8 (5.3), (59.2–74.0)	43.9 (1.5), (41.9–45.5)	41 (27), (5–81)
9	5	290 (9), (269–345)	9.2 (2.0), (6.6–11.5)	69.3 (3.9), (64.5–73.5)	45.6 (1.7), (43.6–47.5)	58 (25), (6–81)
12	5	379 (28), (358–429)	10.3 (2.0), (7.6–12.8)	74.8 (4.0), (69.0–80.2)	46.4 (1.23), (44.5–47.9)	56 (35), (1–90)

Means, standard deviations, and range of scores displayed. AIMS = Alberta Infant Motor Scale.

**Table 2:** Developmental Outcomes.

Infant	ADOS Score (18 m)	ADOS Outcome (18 m)	ADOS Score (36 m)	ADOS Outcome (36 m)	MSEL ELC (36 m)	VABS ABC (36 m)
HR1	17	Autism (moderate-severe concern)	9	Autism	118	86
HR2	18	Autism (moderate-severe concern)	12	Autism	76	70
HR3	3	Little to no concern	6	Non-spectrum	113	101
HR4	12	Mild-moderate concern	4	Non-spectrum	138	82
HR5	1	Little to no concern	0	Non-Spectrum	116	104

HR indicates high risk infant. ADOS indicates the Autism Diagnostic Observation Schedule, MSEL indicates the Mullen Scales of Early Learning, ELC indicates the Early Learning Composite, VABS indicates the Vineland Adaptive Behavioral Scale-II, and ABC indicates Adaptive Behavior Composite.

## Data Availability

The data presented in this study are available on request from the corresponding author. The data are not publicly available due to ethical reasons.
